# How to Safeguard University Students Against HIV Transmission? Results of a Cross-Sectional Study in Southern Italy

**DOI:** 10.3389/fmed.2022.903596

**Published:** 2022-06-24

**Authors:** Francesca Licata, Silvia Angelillo, Antonella Oliverio, Gianfranco Di Gennaro, Aida Bianco

**Affiliations:** Department of Health Sciences, School of Medicine, University of Catanzaro “Magna Græcia”, Catanzaro, Italy

**Keywords:** HIV infection, Italy, prevention, transmission, university students, sexual behaviors

## Abstract

**Introduction:**

Young people represent a growing share of the group living with HIV, and monitoring the sexual behaviors of this vulnerable age group is necessary to control and prevent the HIV/AIDS pandemic. The present study has been conducted to investigate the level of knowledge and attitudes toward HIV infection and sexual behaviors among a sample of undergraduate university students in Southern Italy.

**Methods:**

Data were collected through an anonymous online questionnaire between 1st to 31st July 2020 and included questions on socio-demographic and sexual history characteristics, knowledge and attitudes toward HIV infection, sexual behaviors, and sources of information about HIV. The eligibility criteria for the study were: age between 18 and 30 years and registered as an undergraduate student at the university.

**Results:**

Among the 1,318 students who completed the survey, 62.5% knew that HIV can be transmitted through blood, vaginal secretions and semen. The overall median knowledge score was 10, and the main determinants of knowledge were being younger and attending to medical or life science majors. Less than half of the students (45.8%) reported that they always wear a condom during any sexual activity. Predictors of consistent condom use were being younger, having a later sexual debut and a good knowledge score.

**Conclusions:**

The study findings showed a not completely satisfactory level of knowledge and unsafe sex practices among university students. These results reiterate the need to tailor HIV prevention strategies among young individuals. Such a change could dispel misconceptions about HIV transmission and prevention that affect risk-taking sexual behaviors. These strategies may ultimately contribute to lessing the effect of HIV/AIDS transmission in Italy.

## Introduction

The human immunodeficiency virus (HIV) and the acquired immunodeficiency syndrome (AIDS) still represent global public health issues and are leading causes of years lived with disability in many countries worldwide (1). The number of people newly diagnosed with HIV has increased over the last decade in European countries, and 1 in 2 people living with HIV is diagnosed late (2). In Italy, the Italian HIV-Surveillance System, established in July 2008 (3), reported that the number of newly diagnosed individuals with HIV increased and peaked in 2012, before gradually decreasing until 2019. The proportion of new HIV diagnoses in the 25–29 year age group has remained quite stable over the past decade, and the highest incidence of the infection was seen in this age group (5,5 new diagnoses per 100,000) during 2020. In Italy, the predominant modes of HIV transmission were sex between men and heterosexual contact, accounting for 45.7% and 42.7% of all the new HIV diagnoses, respectively (4).

Young people represent a growing proportion of the group living with HIV (5), and unprotected sex is the most common route of HIV infection in this age group (6). Doubtless, primary prevention measures of HIV infection play an important role in its control and condom use remains the most effective and economical preventive measure (7). Furthermore, U.S. Food and Drugs Administration approved HIV pre-exposure prophylaxis (PrEP) (8) as an additional strategy to prevent HIV acquisition among high-risk individuals (e.g., individuals who have infrequent condom use with one or more partners of unknown HIV status) (9). When taken as prescribed, PrEP provides >90% protection against acquiring HIV (9). Moreover, improvement in testing access and practices also needs immediate attention, since delayed testing places infected individuals at risk of receiving delayed antiretroviral therapy (10) that has proven to be one of the most remarkable public health measures in reducing morbidity and mortality caused by HIV (11). Access to HIV testing and subsequent treatment are also able to decrease the risk of new infections since HIV individuals virally suppressed have no risk of transmitting the virus to HIV-negative sex partners (12).

The reasons young individuals engage in unsafe sex practices despite knowledge of their consequences, continue to serve as an area of research interest, and monitoring the risk perception and sexual behaviors of this vulnerable age group is still necessary to control and prevent HIV/AIDS pandemic. Many studies have described HIV-related knowledge, attitudes, sexual behaviors (13–15) and HIV testing (16, 17) among university students, while a few studies have examined the factors associated with safe sexual behaviors in the Italian setting (13, 15). Therefore, determinants associated with awareness of the risk of exposure to HIV and consistent condom use need to be better understood to facilitate discussion about prevention of HIV among sexually active young individuals in Italy. Thus, the present study has been conducted to investigate the level of knowledge and attitudes toward HIV infection and sexual behaviors among a sample of undergraduate university students in Southern part of Italy. A secondary aim was to test the hypothesis that socio-demographic characteristics and the type of academic major attended may influence the level of knowledge and the risk perception of HIV infection, that in turn, may predict safe sexual behaviors.

## Materials and Methods

### Study Design

This cross-sectional questionnaire-based study was conducted between 1st to 31st July 2020. All students aged between 18 and 30 years attending majors at the “Magna Græcia” University of Catanzaro, Southern part of Italy, were invited to participate.

### Participant Recruitment and Sampling

Prior to data collection, the university administration was approached for approval on data collection. After the approval, data were collected anonymously using an online self-administered questionnaire that was developed based on previous similar studies (13–15, 18). The inclusion criteria required the participants to be undergraduate students and to have voluntarily participated. The exclusion criteria included being under 18 and over 30 years of age. We hypothesized that the academic major could have an effect on the students' knowledge and attitudes toward HIV infection and safe sexual behaviors, so we conducted a stratified sampling. Hence, we decided to include at least 25 males and 25 females from each one of the 28 university majors and based on these assumptions a sample of 1,400 was sought. All the eligible students in a sampling frame were sent to the institutional email address the link to the online questionnaire (achieved using a Google Forms® online application). Afterwards, the questionnaires where more than 40% data are missing in important variables (i.e., sexual behaviors and attitudes toward sexual activity) were excluded from the study (no. 82). The first page of the questionnaire included a brief description of the study aims so that participants could make an informed choice about whether or not to participate. All participants who selected the “agree” option were directed to complete the self-administered questionnaire. To reduce potential repeat responses, the questionnaire could only be submitted once. All participants were assured that there were no identifiers that link students to the survey and they were informed that they could withdraw their participation at any time. Participants were not offered compensation for their time to respond. The research team handled all participants' recruitment, data collection, and the consent process without any interference from the university officers.

### Questionnaire Design

The first version of the questionnaire was pilot tested among a convenience sample of 20 students prior to the start of the study to evaluate the clarity of the items and to estimate the comprehensibility. Data from the pilot study were not included in the final analysis. An online survey was launched on July 1st and remained open until July 31. The survey, which took approximately 15 min to complete, included 27 questions on: (1) socio-demographic and sexual history characteristics; (2) knowledge related to HIV infection (16 questions with a “true/false” response format); (3) attitudes toward HIV infection (3 items on a five-point Likert scale response format); (4) sexual behaviors (2 items on a five-point Likert scale response format); and (5) sources of information about HIV. A total score on knowledge was calculated using all the knowledge questions about HIV transmission modes, strategies of prevention and HIV testing by assigning 1 point to each correct answer, and 0 to an incorrect unknown answer. The total knowledge score ranged from 0 to 16 with higher scores indicated better knowledge. The questionnaire is available as Supplementary Material.

The Local Human Research Ethics Committee approved the study protocol, the participant information sheet, the informed consent form, and the survey questionnaire (ID No. 102/2021703/18).

### Statistical Analysis

Descriptive statistics were presented using means, medians, interquartile range (IQR), and standard deviations (SD) for continuous variables and frequencies for categorical variables. Inferential analyses were conducted to investigate the variables associated with the outcomes of interest. The knowledge score about HIV infection was analyzed through an ordinal regression model (Model 1). Multiple logistic regression models have also been developed to investigate the relationship between the independent variables and the following outcomes of interest: belief that early sexual debut carries an increased risk of HIV transmission (Model 2); belief that individuals with HIV are discriminated and stigmatized by people at large (Model 3); and always wear a condom during any sexual activity (Model 4). The following selected independent variables were included in all models: gender (male = 0; female = 1), age, in years (continuous), major attended (three categories: medical or life science = 0; social science = 1; technology = 2) and need for further information on HIV infection (no = 0; yes = 1). The variable knowledge score about HIV infection (ordinal) was also included in Models 2 and 3. The variables: age of first sexual intercourse (continuous), belief that early sexual debut carries an increased risk of HIV transmission (no = 0; yes = 1), belief that having multiple sex partners increases the chances of acquiring HIV (no = 0; yes = 1) and belief that individuals with HIV are discriminated and stigmatized by people at large (no = 0; yes = 1) were also included in Model 3. In Model 4, the students who have never had sexual intercourse (no. 221) were excluded from the analysis. The goodness of fit of the logistic regressions was ascertained through the Hosmer and Lemeshow test. The statistical significance level was set at a *p*-value ≤ 0.05. Adjusted odds ratio (OR) and 95% confidence interval (CI) were calculated. The data were analyzed using STATA software, version 16.1 (19). The data set was deposited in Mendeley Data repository (doi: 10.17632/vtvnz52wgs.1).

## Results

### Socio-Demographic and Sexual History Characteristics of the Participants

Among the 1,318 students who completed the survey, more than two-thirds (70.3%) were female and the median age was 23 years (IQR: 21–25 years). Of all participants, 58.3% were enrolled in medical or life science courses. Regarding sexual history, 221 (16.8%) of the students reported they have never had sexual intercourse; among those who have had sexual intercourse, the mean age of the sexual debut was 17.5 years (SD ± 2.2), and 15.2% had experienced an early debut, at or before age 14 years.

### Knowledge Regarding HIV Transmission

Table 1 presents the answers to the knowledge statements about HIV infection. Less than one-third (28.8%) knew that in Italy new cases of HIV infection are mainly attributable to heterosexual intercourse, in accordance with surveillance data during the study period. Regarding the modes of transmission, only 62.5% of the subjects were knowledgeable that HIV can be transmitted through certain body fluids such as blood, vaginal secretions and semen. In addition, 31.4% and 86% of participants knew that the virus can not be transmitted by sharing a razor/toothbrush or through saliva, respectively. Two-third of the sample (66%) believed that it is necessary to avoid unprotected sexual intercourse with an unknown partner to prevent HIV transmission.

**Table 1:** Respondents' knowledge related to HIV infection.

**Statements (1,318)**	**Correct**	
	***N***	**%**
**In Italy new cases of HIV infection are mainly attributable to heterosexual intercourse** (true)	380	28.8
**HIV can be transmitted** ***via***		
Blood (true)	1,231	93.4
Sexual contact (i.e. vaginal secretion and semen) (true)	1,103	83.7
Saliva (false)	1,133	86
Sharing a razor or toothbrush (false)	414	31.4
Sharing toilets (false)	1,098	83.3
Open cuts or sores in the skin (true)	1,252	95
**HIV transmission could be prevented by**		
Consistent condom use (true)	1,200	91.1
“Femidon” (true)	570	43.3
Sexual intercourse with someone of know HIV status (true)	870	66
Sexual intercourse only with healthy people (false)	1,057	80.2
Vaccine for HIV before having risky relationships (false)	1,098	83.3
**HIV testing**		
Has to be prescribed by a physician (false)	551	41.8
Is provided to anyone free of charge (true)	729	55.3
**An individual could experience early symptoms of the acute primary infection within 2 to 4 weeks after infection with HIV** (true)	249	18.9
**The time between when a person may have been exposed to HIV and seroconversion (i.e. window period) usually ranged from 20 to 90 days** (true)	407	30.9

### Knowledge Regarding HIV Prevention and Testing

Regarding knowledge about strategies to prevent HIV infection, the vast majority of the students (91.1%) were aware that always wearing a condom during any sexual activity can prevent the sexual transmission of HIV, but only 43.3% knew that femidon is a female condom. In addition, less than one-third (30.9%) knew that the “window period” (i.e., the time between exposure to HIV and when the antibodies can be reliably detected from a standard HIV test) usually ranged from 20 to 90 days (Table 1).

### Ordinal Regression Analysis Determining Factors Associated With Knowledge Score

The overall median knowledge score was 10 (IQR: 9–11), and the results of the ordinal regression model showed that the knowledge score significantly increased with every year of age (coeff: 0.07; 95% CI: 0.04–0.11). A lower level of knowledge was observed in students attending social science (coeff: −1.01, 95% CI: −1.24–0.78) or technology majors (coeff: −0.55, 95% CI: −0.84–0.26) compared with those attending medical or life science majors and in those who needed for further information on HIV infection (coeff: −0.33, 95% CI: −0.55–0.11) (Model 1 in Table 2).

**Table 2:** Results of the regression models for potential determinants of the outcomes of interest.

**Model 1. Outcome: Knowledge score about HIV infection** **Log likelihood = − 2480.1943; Prob > chi2 < 0.001; Obs = 1,318**			
**Variables**	**Coefficient**	**95% CI**	***p***
**Majors**			
Medical or life science^*^	1.00		
Social science	−1.01	−1.24–0.78	<0.001
Technology	−0.55	−0.84–0.26	<0.001
**Age, continuous**	0.07	0.04–0.11	<0.001
**Need further information on HIV infection**			
No^*^	1.00		
Yes	−0.33	−0.55–0.11	0.003
**Gender**			
Male^*^	1.00		
Female	0.07	−0.14–0.28	0.494
**Model 2. Outcome: Belief that early sexual activity carries an increased risk of HIV transmission** **Log likelihood = −846.41204 Prob > chi2 < 0.001; Obs = 1,318**			
**Variables**	**OR**	**95% CI**	***p***
**Majors**			
Medical or life science^*^	1.00		
Social science	0.34	0.26–0.46	<0.001
Technology	0.59	0.42–0.83	0.002
**Age, continuous**	1.07	1.03–1.12	0.001
**Knowledge score on HIV infection**	0.98	0.92–1.04	0.425
**Gender**			
Male^*^	1.00		
Female	0.92	0.72–1.18	0.506
**Need further information on HIV infection**			
No^*^	1.00		
Yes	1.04	0.80–1.35	0.776
**Model 3: Outcome: Belief that individuals with HIV are discriminated and stigmatized by people at large** **Log likelihood = −787.31336; Prob > chi2 < 0.001; Obs = 1,318**			
**Variables**	**OR**	**95% CI**	***p***
**Majors**			
Medical or life science^*^	1.00		
Social science	0.86	0.64–1.14	0.289
Technology	0.57	0.41–0.81	0.001
**Age, continuous**	1.06	1.01–1.11	0.010
**Knowledge score on HIV infection**	1.07	1.01–1.14	0.030
**Need further information on HIV infection**			
No^*^	1.00		
Yes	1.13	0.86–1.48	0.393
**Gender**			
Male^*^	1.00		
Female	1.09	0.84–1.42	0.510
**Model 4. Outcome: Always wear a condom during any sexual activity** **Log likelihood = −725.87664; Prob > chi2 < 0.001; Obs = 1,097** ^**#**^			
**Variables**	**OR**	**95% CI**	***p***
**Age of first sexual intercourse, continuous**	1.21	1.14–1.28	<0.001
**Age, continuous**	0.92	0.88–0.96	<0.001
**Knowledge score on HIV infection**	1.09	1.02–1.16	0.013
**Gender**			
Male^*^	1.00		
Female	0.79	0.60–1.03	0.081
**Need further information on HIV infection**			
No^*^	1.00		
Yes	1.22	0.92–1.62	0.174
**Belief that individuals with HIV are discriminated and stigmatized by people at large**			
No^*^	1.00		
Yes	0.84	0.64–1.10	0.197
**Majors**			
Medical or life science^*^	1.00		
Social science	1.22	0.90–1.66	0.206
Technology	1.20	0.83–1.72	0.334
**Belief that having multiple sex partners increases the chances of acquiring HIV**			
No^*^	1.00		
Yes	0.92	0.62–1.37	0.681
**Belief that early sexual activity carries an increased risk of HIV transmission**			
No^*^	1.00		
Yes	0.97	0.75–1.26	0.837

* *Reference category*.

# *Observations included those who had their sexual debut*.

### Attitudes Toward Risk of HIV Infection and Multiple Logistic Regression Analysis

Respondents' attitudes toward HIV infection are detailed in Table 3. More than three-quarters (78.8%) of participants strongly agreed that having multiple sex partners increases the chances of acquiring HIV. Additionally, 4 out of 10 (39.4%) participants affirmed that early sexual debut carries an increased risk of HIV transmission, and older students (OR: 1.07; 95% CI: 1.03-1.12) were more likely to perceive it as a risk factor (Model 2 in Table 2). Furthermore, the results of the model predicting this positive attitude showed an independent negative association of attending social science (OR: 0.34; 95% CI: 0.26-0.46) or technology (OR: 0.59; 95% CI: 0.42-0.83) majors compared with attending medical or life science.

**Table 3:** Respondents' attitudes toward HIV infection and self-reported sexual behaviors.

**Attitudes (1,318)**	**Strongly agree/Agree**		**Uncertain**		**Strongly disagree/Disagree**					
	***N***	**%**	***N***	**%**	***N***	**%**				
Early sexual activity carries an increased risk of HIV transmission	**519**	**39.4**	296	22.4	503	38.2				
Having multiple sex partners increases the chances of acquiring HIV	**1,037**	**78.7**	137	10.4	144	10.9				
Individuals with HIV are discriminated and stigmatized by people at large	**925**	**70.2**	240	18.2	153	11.6				
Behaviors (1,097)^*^	**Never**		**Rare**		**Occasionally**		**Often**		**Always**	
	***N***	**%**	***N***	**%**	***N***	**%**	***N***	**%**	***N***	**%**
Use of condom during any sexual intercourse	134	12.2	106	9.6	150	13.7	205	18.7	502	45.8
Alcohol consumption before having sexual intercourse	374	34.1	307	28	343	31.3	66	6	7	0.6

*Number and percentages referring to positive answers are in bold*.

* *Observations included those who had their sexual debut*.

### Attitudes Toward the HIV Stigma and Multiple Logistic Regression Analysis

More than two-thirds (70.2%) of the participants believed that individuals with HIV are discriminated and stigmatized by people at large (Table 3). The results of the multiple logistic regression analysis indicated that with every one-point increase in knowledge score about HIV infection (OR: 1.07; 95% CI: 1.01-1.14) and every 1 year increase of age (OR: 1.06; 95% CI: 1.01–1.11), the odds of believing that individuals with HIV are stigmatized resulted in a 7% and 6% increase, respectively. Furthermore, attending technology majors (OR: 0.57; 95% CI: 0.41–0.81) compared with attending medical or life science majors was a significant predictor of having that positive attitude (Model 3 in Table 2).

### Self-Reported Sexual Behaviors and Multiple Logistic Regression Analysis

Respondents' self-reported sexual behaviors are showed in Table 3. Regarding sexual behaviors, less than half of the sample (45.8%) reported that they always wear a condom. Results of the multiple logistic regression analysis showed that being younger (OR: 0.92; 95% CI: 0.88–0.96) and a later sexual debut (OR: 1.21; 95% CI: 1.14–1.28) were predictors of consistent use of condoms. Moreover, for a one-point increase of the knowledge score, the odds of consistent condom use increases by 1.09 times (OR: 1.09; 95% CI: 1.02–1.16) (Model 4 in Table 3). The Hosmer and Lemeshow test indicates satisfactory goodness of fit of the model (*p* = 1.00).

### Sources of Information

The main sources of information used to learn about HIV were mass media (56.7%), followed by University (55.1%), social networks (47.8%) and government websites/international organizations (41.6%). However, almost three-quarters (73.6%) of the participants wished to receive additional information.

## Discussion

The findings of the present survey provide an up-to-date insight about knowledge, attitudes, and sexual behaviors of undergraduate university students regarding HIV infection in order to identify opportunities to improve adherence to prevention strategies through future tailored interventions. The understanding of factors unique to this age group is pivotal to aid in the design of the educational campaigns, considering that HIV infection constitutes a societal threat with a devastating impact among young people due to their lack of effective maturity to make beneficial sexual decisions (20).

This survey provides 3 major findings. First, the results indicate that the level of knowledge reported by study participants was not completely satisfactory concerning the general questions about HIV infection. More than half of the participants did not know about the femidom, and this finding is in line with a previous survey performed by the Italian Ministry of Health among women (21). Furthermore, the students were not fully knowledgeable about the importance of avoiding unprotected sexual intercourse with an unknown partner and about adequate HIV testing. Especially in the youngest age groups, one of the most concerning ways of transmission is occasional sex (22), and the finding that only one-third of the sample recognized unprotected sexual intercourse with an unknown partner as a risk factor of HIV infection, is of concern. Even more worrying is the lower proportion of students knowledgeable about the window period of HIV infection. Before seroconversion, there may not be detectable levels of HIV antibodies in a person's blood, and a blood test could produce a false negative result. A positive HIV antibody test will not appear until the body makes enough HIV antibodies to be detected. Individual who received negative HIV result during this period perceived themselves as being at low risk of acquiring HIV (23). On the other hand, during the window period, a person may develop symptoms similar to the flu lasting from a few days to a few weeks and less than one-fifth were knowledgeable about this information. The lack of this knowledge could negatively affect the perception of real risk of HIV infection and, ultimately, an appropriate HIV testing behavior. As expected, students attending medical or life science majors were significantly more knowledgeable than those attending other majors. As a high-knowledge group, those students can spread their knowledge to the individuals around them. It is important to develop health literacy skills early in life, and the promotion of health literacy for young people could be delivered also in education, as well as in the health sector.

The second key result is that only 3 out of 10 students were knowledgeable that in Italy new cases of HIV infection are mainly attributable to heterosexual intercourse. Although the current surveillance data (4) showed that new HIV diagnoses are mainly attributable to men who have sex with men (MSM), data during the study period (i.e., 1–31st July 2020) indicated as predominant heterosexual transmission (24). The wrong notion that HIV is a health problem mainly of MSM group could generate the self-perception of a low risk of HIV infection. In addition to the latter, the belief that individuals with HIV are discriminated and stigmatized by people at large (reported from 70.2% of the participants) could act as determinants of a strong delay in HIV testing (25). Moreover, since a certain proportion of MSM have heterosexual behaviors and HIV prevalence in this group is also high, adequate attention should be given to sexual behaviors, regardless of sexual orientation. Although HIV infection has been linked with homosexuality ever since it appeared, studies showed that sexual orientation is relatively unimportant in the transmission of HIV (26). Indeed, AIDS is becoming more and more a heterosexual phenomenon, so that it is pivotal to focus upon all types of sexual behaviors in order to prevent HIV infection.

Thirdly, although the vast majority of the students (91.1%) correctly answered the question about the effectiveness of consistent condom use in preventing the sexual transmission of HIV, less than half of the sample (45.8%) reported always using a condom during any sexual intercourse. This figure is of concern considering that consistent and correct condom use was one of the earliest recommendations for preventing HIV infection at the start of the pandemic outbreak. It is well known that condoms are inexpensive and offer strong protection against transmission of HIV and other sexually transmitted infections, especially among university students who are a group constantly exposed to sexual risk behaviors that make them more vulnerable to HIV infection (27, 28). In addition, students are a very mobile group, and they can become dissemination reservoirs for the spread of HIV in society (29). Against this scenery, it becomes critical that effective HIV and AIDS intervention strategies among university students are implemented, effectively promoted and evaluated. The association between two safe sexual behaviors (later sexual debut and condom use) could suggests that healthy and protective sexual habits can cluster together.

## Limitations

To appreciate this study, some limitations must be acknowledged. First and foremost, the relationship between the predictor variables and the dependent variables should not be taken as the cause-and-effect relationship, since this study has a cross-sectional design. However, the present research is able to describe general associations. Second, the study findings are derived from self-reported data and it is possible that respondents may over-report socially desirable attitudes or behaviors or under-report socially undesirable attitudes or behaviors, which may have affected the reliability of the results. Nevertheless, it was demonstrated that the means for improving the solidity of self-reported data have to include adherence to procedures that maximize anonymity and confidentiality, as we performed in the survey. Third, the study was restricted to university students from one region and one must be cautious in interpreting the results as generalizable for students or young adults in the whole country. However, it is reasonable to suppose that the findings of the study may represent at least the university students of the regions of Southern Italy. Fourth, another possible limitation, as tends to happen in online surveys, is that the participants could look up the correct answers online or confer with someone before responding; nevertheless, the correct answer rate was similar to that of a previous study (18), so we are confident that this is not an issue in our data. Lastly, the meaning of “sexual intercourse” was not specified in the questionnaire, and misunderstanding about it might be happened. One of the study aim was to investigate safe sexual behaviors, such as consistent condom use for every act of vaginal, anal and oral sex, so it could be argued that an eventual misunderstanding about “sexual intercourse” might have generated an underestimation of the actual prevalence of unprotected sex.

## Conclusion

The study findings showed a not completely satisfactory level of knowledge and unsafe sex practices among university students. These results reiterate the need to tailor HIV prevention strategies. Such a change could dispel misconceptions about HIV transmission and prevention that affect risk-taking sexual behaviors (30). These strategies may ultimately contribute to lessening the effect of HIV/AIDS transmission in Italy.

## Data Availability Statement

The dataset was deposited in Mendeley Data repository (https://doi.org/10.17632/vtvnz52wgs.1).

## Ethics Statement

The Calabria Centre Local Human Research Ethics Committee approved the study protocol, the participant information sheet, the informed consent form, and the survey questionnaire (ID No. 102/2021703/18). The participants provided their written informed consent to participate in this study.

## Author Contributions

FL and AO participated in the conceptualization, design of the study, and contributed to the data collection. FL, SA, and GDG contributed to the data analysis, interpretation, and preparation of the first draft of the manuscript. AB the principal investigator, designed the study, coordinated and supervised data collection, was responsible for the statistical analysis and interpretation, and wrote the final article. All authors have given final approval of the version to be published and agreed to be accountable for all aspects of the work.

## Conflict of Interest

The authors declare that the research was conducted in the absence of any commercial or financial relationships that could be construed as a potential conflict of interest.

## Publisher's Note

All claims expressed in this article are solely those of the authors and do not necessarily represent those of their affiliated organizations, or those of the publisher, the editors and the reviewers. Any product that may be evaluated in this article, or claim that may be made by its manufacturer, is not guaranteed or endorsed by the publisher.
