# Supplementary material for: How to Safeguard University Students Against HIV Transmission? Results of a Cross-Sectional Study in Southern Italy
Source: Front Med (Lausanne). 2022 Jun 24;9:903596. doi: 10.3389/fmed.2022.903596 (PMC9263725; doi:10.3389/fmed.2022.903596)
Supplement: Supplementary file 1 [file Data_Sheet_1.DOCX]

**QUESTIONNAIRE**

**HOW TO SAFEGUARD UNIVERSITY STUDENTS AGAINST HIV TRANSMISSION?**

The purposes of the survey are to evaluate the level of knowledge and attitudes towards HIV infection and sexual behaviors. According to General Data Protection Regulation (EU) 2016/679, data will be analyzed only in an aggregate way and there will be no nominal data spreading. Precautions have been taken in order to guarantee confidentiality of gathered data and anonymity on respondents, so you can feel completely free to respond.

Agreement to participate in the survey: □Yes □No

Please, mark with an “X” or write after the question where appropriate

**A.** **SOCIO-DEMOGRAPHIC CHARACTERISTICS**

**This section is designed to gather information about your socio-demographic characteristics**

A.1 How old were you on your last birthday? ________

A.2 What is your gender? □ Male □ Female

A.3 What course do you attend at University?________________________

A.4 Have you ever had sexual intercourse? □Yes □No (skip to question B1)

A.5 How old were you when you had sexual intercourse for the first time? __________

**B. KNOWLEDGE ABOUT HIV INFECTION**

|  | **True** | **False** | **Do not know** |
| --- | --- | --- | --- |
| B.1 In Italy new cases of HIV infection are mainly attributable to heterosexual intercourse | □ | □ | □ |
| B.2 HIV can be transmitted via: |  |  |  |
| blood | □ | □ | □ |
| sexual contact (vaginal secretion and semen) | □ | □ | □ |
| saliva | □ | □ | □ |
| sharing a razor or toothbrush | □ | □ | □ |
| sharing toilets | □ | □ | □ |
| open cuts or sores in the skin | □ | □ | □ |
| B.3 HIV transmission could be prevent by: |  |  |  |
| consistent condom use | □ | □ | □ |
| femidon | □ | □ | □ |
| sexual intercourse with someone of know HIV status | □ | □ | □ |
| sexual intercourse only with healthy people | □ | □ | □ |
| vaccine for HIV before having risky relationships | □ | □ | □ |
| B.4 HIV testing: |  |  |  |
| has to be prescribed by a physician | □ | □ | □ |
| is provided to anyone free of charge | □ | □ | □ |
| B.5 An individual could experience early symptoms of the acute primary infection within 2 to 4 weeks after infection with HIV | □ | □ | □ |
| B.6 The time between when a person may have been exposed to HIV and seroconversion (i.e. window period) usually ranged from 20 to 90 days | □ | □ | □ |

**C. ATTITUDES TOWARDS HIV INFECTION**

**In general, do you believe that:**

|  | **Strongly agree** | **Agree** | **Uncertain** | **Strongly disagree** | **Disagree** |
| --- | --- | --- | --- | --- | --- |
| C.1 Early sexual activity carries an increased risk of HIV transmission | □ | □ | □ | □ | □ |
| C.2 Having multiple sex partners increases the chances of acquiring HIV | □ | □ | □ | □ | □ |
| C.3 Individuals with HIV are discriminated and stigmatized by people at large | □ | □ | □ | □ | □ |

**D. SEXUAL BEHAVIORS (skip to section E if you have never had sexual intercourse)**

|  | **Never** | **Rare** | **Occasionally** | **Often** | **Always** |
| --- | --- | --- | --- | --- | --- |
| D.1 Do you use a condom during sexual intercourse? | □ | □ | □ | □ | □ |
| D.2 Did you happen to take alcohol before you having sexual intercourse? | □ | □ | □ | □ | □ |

**E.** **SOURCES OF INFORMATION**

**This section is designed to explore the sources from which you acquire information regarding HIV infection**

| E.1 From which of the following sources do you acquire information about HIV infection? (multiple answers allowed)  Mass media (tv, radio, newspaper) Social network (facebook, twitter, blog, etc)  Family Friends Physician University None  Government websites/international organization Other, please specify_____ |
| --- |
| E.2 Do you need further information about HIV infection? Yes No I don’t know |

*Thanks for answering our questionnaire.*

*If you would like to add something more, please write it down in the space below:*

*---------------------------------------------------------------------------------------------------------------------------------------------------------------------------------------------------------------------------------------------------------------------------------------------------------------------------------------------------------------------------------------------------------------------------------------------------------------------------------------------------------*
